# Surveillance of Zoonotic Pathogens in Small Mammals Across Forests With Different Levels of Anthropization in Eastern France

**DOI:** 10.1155/tbed/4038648

**Published:** 2026-05-03

**Authors:** Marie Bouilloud, Maxime Galan, Anaïs Bordes, Caroline Tatard, Philippe Gauthier, Julien Pradel, Guillaume Castel, Hussein Alburkat, Lara Dutra, Tarja Sironen, Clémence Galon, Sara Moutailler, Zorée Djelouadji, Benjamin Roche, Nathalie Charbonnel

**Affiliations:** ^1^ CBGP, INRAE, IRD, CIRAD, Institut Agro, University of Montpellier, Montpellier, France, umontpellier.fr; ^2^ Cornell University, Ithaca, New York, USA, cornell.edu; ^3^ Veterinary Medicine, University of Helsinki, Helsinki, Finland, helsinki.fi; ^4^ Faculty of Medicine, Al-Muthanna University, Muthanna, 6600, Iraq, uoalmuthana.edu.iq; ^5^ Norwegian College of Fishery Science, UiT The Arctic University of Norway, Tromsø, Norway, uit.no; ^6^ ANSES, INRAE, Ecole Nationale Vétérinaire d’Alfort, UMR BIPAR, Laboratoire de Santé Animale, Maisons-Alfort, F-94700, France, vet-alfort.fr; ^7^ USC 1233 RS2GP, VetAgro Sup, University of Lyon, Marcy L’Etoile, France, univ-lyon1.fr; ^8^ MIVEGEC, IRD, CNRS, University of Montpellier, Montpellier, France, umontpellier.fr

**Keywords:** forests, public health, urban parks, wildlife, zoonoses

## Abstract

The emergence of infectious diseases associated with land‐use changes is well‐documented. However, the presence and dynamics of zoonotic pathogens in small mammals within European forests, whether from rural development or urban greening, remain underexplored. To describe zoonotic hazards in these ecosystems, and to assess the influence of biotic and abiotic factors on their distribution, we analyzed 1549 individuals from 18 small mammal species sampled across forest types representing different levels of anthropization using both targeted and broad‐spectrum serological and molecular methods. We detected nine bacteria and five Apicomplexa that are potentially pathogenic to humans. Zoonotic pathogen richness and community composition varied significantly across host species, sites, and sampling periods. Richness was lower in forested urban parks, possibly due to the absence of vectors or intermediate hosts within cities. It was higher in urban adapter species, even within a given forested habitat, emphasizing the important role of specific life‐history traits. Pathogen community structure was shaped by forest anthropization and host ecology, with marked differences between urban and rural forested environments and between urban adapter and dweller species within forested urban parks. The seroprevalence of key pathogens (e.g., *Bartonella*, *Orthopoxvirus*, *Neoehrlichia mikurensis*, and Sarcocystidae) showed spatial, temporal, and host‐specific variation. Epidemiological differences between sites often exceeded those between habitat types, in particular when comparing protected and managed forests, highlighting the importance of local ecological context. Nevertheless, some patterns reflected the influence of forest anthropization and species urban adaptation strategies for certain zoonotic agents. High anthropization in forests was associated with elevated *Bartonella* prevalence, driven by urban‐adapter species rather than forest dwellers, emphasizing local ecological interactions between hosts and pathogens. Besides, higher levels of *Orthopoxvirus* seroprevalence were associated with adapter species in protected forests where they might be more abundant. Altogether, these findings underscore the need for integrated and multipathogen wildlife surveillance to anticipate and mitigate disease risks at the human–environment–animal interface.

## 1. Introduction

The recent rise in the number and frequency of zoonotic diseases reemerging worldwide is still concerning [1]. While the causes of this rise are complex and multifaceted, land‐use changes such as deforestation and urbanization seem to play a critical role. They significantly impact environments and host communities, which may result in new favorable conditions for interspecies transmission of pathogens and human exposure to zoonotic agents [2–4].

Forests in Europe are essential and vulnerable ecosystems that host a substantial proportion of terrestrial biodiversity, providing habitats for a wide variety of plant and animal species [5]. They are important interfaces between wildlife, domestic animals, and humans, creating opportunities for pathogen transmission [6]. In Europe, these interactions are amplified by the growing frequency of recreational activities near residential areas [7], but also by ecological pressure that lead to habitat disruption and fragmentation, which displaces wildlife and forces closer contact with humans and domestic animals [4, 8].

Despite these growing risks of zoonotic pathogen transmission in European forests, wildlife surveillance remains limited, often focusing on a single known species and pathogen, mainly in response to outbreaks [9]. Therefore, it is crucial to implement proactive monitoring of wildlife and zoonotic pathogens to anticipate potential emerging and reemerging zoonotic diseases, and adapt existing prevention policies to better inform, raise awareness and prepare residents, professionals, and recreational users [10].

As reservoirs of pathogens and bridge species between wildlife and humans [11–13], small mammals constitute a relevant model for pathogen surveillance, positioning them as effective sentinels of zoonotic hazards. However not all small mammal species contribute equally to pathogen transmission. Small mammal species exhibit varying degrees of competence, that is, capacity to maintain and transmit pathogens [11, 14]. Landscape anthropization can further accentuate the role of species with particular ecological strategies, especially generalist (adapter) or synanthropic (urban dweller) species, by reshaping community composition and selectively favoring taxa that tolerate or benefit from human disturbance [15]. For example, long‐term urbanization in Central China has favored the striped field mouse, leading to its proliferation at the expense of other rodent species and increasing its importance as a reservoir of Hantaan virus [15]. More globally, the meta‐analysis described by Gibb et al. [3] showed that anthropization and urbanization lead to a significantly higher proportion of known zoonotic hosts, although their dataset lacked robust representation from urban environments. Albery et al. [16] further found that urban‐adapted mammals host a higher number of zoonotic parasites, although this pattern may partly reflect research bias rather than intrinsic ecological mechanisms. In contrast, urban avoider species tend to decline or even become locally extinct under increasing anthropogenic pressure [17, 18]. Altogether, these shifts in small mammal community composition associated with anthropization can drive changes in host–pathogen dynamics, influencing both the maintenance of pathogens within wildlife populations and the likelihood of spillover to humans [19, 20]. From an Ecohealth perspective, monitoring small mammals and their pathogens, in a context of increased landscape anthropization, may be critical to evaluate zoonotic hazards. It could help identifying both the host species most likely to act as pathogen reservoirs and the habitats where transmission and spillover risk are highest.

To address this issue, we surveyed small mammals and their zoonotic pathogens across forested areas of Eastern France representing different levels of anthropization, covering urban and rural areas [21]. We pursued two complementary objectives. First, our objective was to provide a comprehensive description of the diversity and composition of small mammal communities and their associated zoonotic pathogens in these forested areas. We anticipated that analyzing a broad range of small mammal species across diverse forested environments, using multiple pathogen detection methods, would reveal that the zoonotic hazard, that is, the presence in the ecosystem of zoonotic pathogens [22], which is typically inferred from human cases or existing wildlife surveys focused on particular diseases, has been significantly underestimated.

Second, we analyzed the influence of host species and sites’ level of forest anthropization on the diversity and composition of zoonotic pathogen communities associated with small mammals. More specifically, we compared host species based on their ecological niche and degree of adaptation to anthropized landscapes, using urban adapter, dweller, and avoider categories [18]. We surveyed forested areas with different levels of management, from rural protected (biological reserve) or managed forests to urban forested parks.

Last, we discussed the implications of these results for wildlife surveillance and the potential need to reconsider prevention policies and guidance on zoonotic hazards. Monitoring small mammals could inform local public health and conservation policies. While exploring these applied aspects was not the primary aim of this study, we showed that our results contribute to the ecological understanding required for such integrative approaches. A better understanding of these hazards provides a foundation for developing strategies to limit human exposure and, consequently, to reduce zoonotic risk, all of which are essential for making scientifically‐informed decisions that support future forest management and urban greening efforts.

## 2. Materials and Methods

All statistical analyses were performed using R v4.4.0 [23].

### 2.1. Small Mammal Sampling

Small mammal sampling was conducted biannually between spring 2020 and spring 2022, in four to six forested sites in Eastern France, encompassing different levels of forest anthropization, which corresponds to the degree of human influence on forest ecosystems, assessed through management intensity, forest fragmentation, and surrounding land use. The study sites represent a clear gradient of anthropization. Less anthropized sites are long‐term unmanaged protected forests (FRFGRI and FRFGLA), where trees experience their full natural life cycle with almost no human perturbation. Then managed forests (FRFCOR and FRFMIG) are exploited for timber production and are fragmented by roads and agricultural land. Finally, urban parks (FRPDLL and FRLTO) are highly modified and disturbed. They include fragmented forested areas and are characterized by intensive management practices, exotic tree plantations, and high levels of human presence and recreational use (Figure 1A). Sampling protocols and study sites are described in detail in Pradel et al. [21]. Spatial and multivariate analyses were conducted to validate the different levels of anthropization across study sites. These analyses confirmed that anthropogenic variables (land use and forest fragmentation metrics) primarily explained differences among sites, thereby validating the robustness of the sampling design (Supporting Information 1: Figure S1).

Figure 1Overview of study sites. (A) Sampling plan. The inset map shows the study region within Europe, with study sites marked in red. The main map provides a zoomed view of this region in France, displaying the six study sites: FRPLTO: Lyon, Park Tête d’Or (Rhône); FRPDLL: Marcy l’Étoile, Domaine Lacroix Laval (Rhône); FRFCOR: Cormaranche‐en‐Bugey (Ain); FRFGRI: Arvière, La Griffe au Diable (Ain); FRFMIG: Mignovillard (Jura); FRFGLA: Esserval‐Tartre, La Glacière (Jura). Each point is surrounded by a thick border reflecting the associated habitat type: dark green for protected forests (FRFGRI and FRFGLA), light green for managed forests (FRFCOR and FRFMIG), and red for urban forested parks (FRPDLL and FRPLTO). *n* indicates the number of small mammals studied per site. (B) Principal coordinate analysis (PCoA) illustrating differences in small mammal community composition across study sites and time periods. Each point represents a unique combination of site and sampling period. Different shapes indicate the sampling periods, ordered chronologically from spring 2020 to spring 2023. Ellipses represent habitat types, using the same color code as in the map. Within each ellipse, pie charts illustrate the average relative abundance of each small mammal species. (Species codes: *Asyl* = *Apodemus sylvaticus*, *Afla* = *Apodemus flavicollis*, *Cgla* = *Clethrionomys* (*Myodes*) *glareolus*, *Crus* = *Crocidura russula*, *Cleu* = *Crocidura leucodon*, *Mmus* = *Mus musculus*, *Rnor* = *Rattus norvegicus*, *Ggli* = *Glis glis*, *Msub* = *Microtus subterraneus*, *Marv* = *Microtus arvalis*, *Magr* = *Microtus agrestis*, *Nfod* = *Neomys fodiens*, *Svul* = *Sciurus vulgaris*, *Eeur* = *Erinaceus europaeus*, *Sara* = *Sorex araneus*, *Scor* = *Sorex coronatus*). Species were grouped into urban adaptation categories: avoiders (light green), which avoid urban environments; adapters (dark green), which occur across all habitat types; dwellers (red), which are exclusively found in urban parks (Supporting Information 1: Figure S2).

**Figure:**
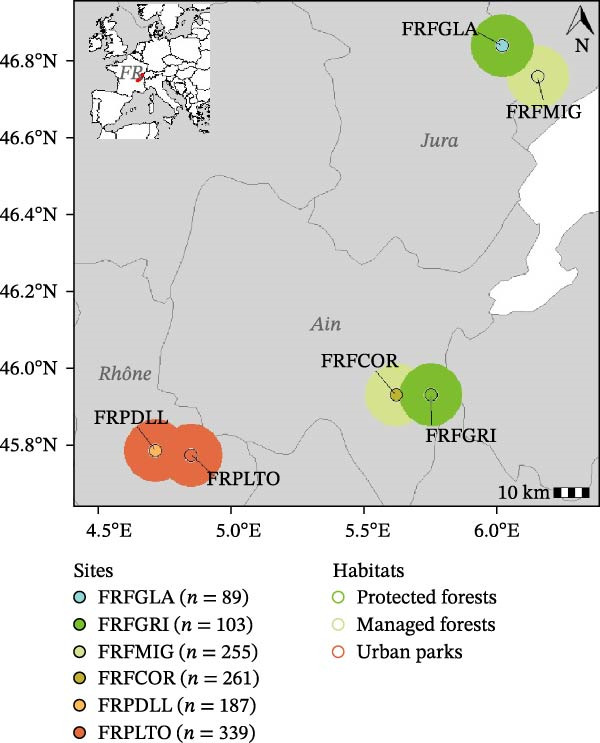
(A)

**Figure:**
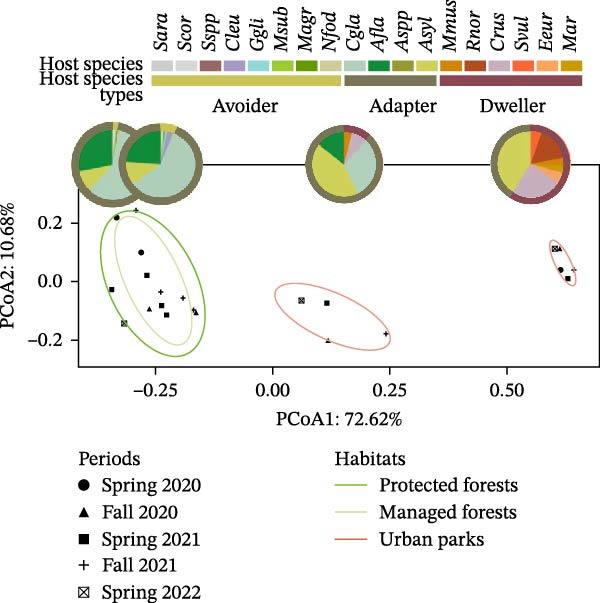
(B)

Animals were euthanized and dissected to collect individual information (weight, length, and reproductive features) and organs necessary for further pathogen detection. All individuals trapped in 2020 were initially sequenced using the COI gene to genetically confirm species identity at each sampling site. Subsequent species identification was performed either morphologically, when reliable, or genetically for cryptic species, namely, *Apodemus* spp. were identified using rapid AP‐PCR, while *Microtus* spp. and insectivores were determined through COI sequencing (see details in Pradel et al. [21]). Individual sex was determined based on sexual morphological traits. Age class (juvenile vs. adult) was assigned according to secondary sexual traits and body mass following [24].

The composition of small mammal communities was analyzed through a Bray–Curtis dissimilarity matrix, generated with the package vegan, and ordered using principal coordinates analysis (PCoA) with the package stats (Supporting Information 1: Figure S2). Based on this analysis and the terminology proposed in Fischer et al. [18], we classified small mammal species according to their urban adaptation: species unable to survive in cities are “avoiders,” those tolerating and thriving in both urban and rural environments are “adapters,” and those living primarily at the expense of humans as “dwellers.”

### 2.2. Zoonotic Pathogen Detection

Pathogen detection was performed to characterize the zoonotic hazard associated with small mammal communities across sites and sampling dates. Based on the literature [25], major rodent‐borne zoonotic pathogens were investigated through targeted screening using serological and qPCR approaches. In addition, a metabarcoding approach was used to identify potentially pathogenic bacteria with no a priori [26].

#### 2.2.1. Serological Analyses for Viruses

Blood samples collected from heart in PBS were tested for IgG antibodies against *Orthohantaviruses* (Puumala virus [PUUV] and Dobrava virus [DOBV]), *Orthopoxviruses* (OPXV), and *Mammarenaviruses* (LCMV) using indirect fluorescent antibody tests IFAT [27, 28]. Positive controls included sera from antibody‐positive bank voles for DOBV and OPXV, sera from PUUV and LCMV antibody‐positive human (Progen, Heidelberg). The secondary antibody was a goat anti‐mouse IgG conjugated with fluorescein (Jackson ImmunoResearch, Pennsylvania, USA) for all assays, but PUUV and LCMV positive controls, for which anti‐human IgG was used.

#### 2.2.2. Detection of Pathogenic *Leptospira* Species

DNA was extracted from the kidney, target organ for leptospires, of small mammal stored in ethanol with Biobasic kit and pathogenic leptospires were detected using quantitative real‐time PCR (qPCR) with Taqman probe [29], targeting the 32‐kDa lipoprotein‐coding gene (LipL32). All DNA extractions were processed in duplicates, with both positive and negative controls. Using LC480 software, individuals with a *C*_T_ < 40 were considered positive for pathogenic *Leptospira* species.

Some of the positive samples from all sampling sites and periods, with lowest *C*_T_, were genotyped to characterize *Leptospira* species and serovars, as described in Garcia‐Lopez et al. [29].

#### 2.2.3. 16S rRNA Metabarcoding for Bacteria Screening

Potential pathogenic bacteria from small mammals were detected in the spleen, an organ involved in pathogen filtration, preserved in ethanol, using the enhanced metabarcoding method of Galan et al. [26]. This approach minimized the risk of false positive results by the validation of the data through systematic technical replicates, filtering thresholds and extensive controls at each step. DNA was extracted using a Qiagen kit, followed by PCR amplification and paired‐end Illumina MiSeq sequencing of the V4 region of the 16S rRNA gene [30]. The sequences were demultiplexed and analyzed using the FROGS (Find Rapidly OTU with Galaxy Solution) pipeline [31]. The sequences were grouped using SWARM [32] with a maximum clustering distance of *d* = 3 and then compared by *BLAST* to the Silva database v138.1 for taxonomic assignment. The resulting abundance tables were filtered to eliminate potential false positives by applying three series of filters described in Galan et al. [26]. The results obtained at the different stages of the processing workflow are available on Zenodo. Taxa corresponding to zoonotic pathogens were selected based on the literature.

#### 2.2.4. Specific Characterization of Bacteria Detected by the 16S rRNA Metabarcoding

We refined taxonomic resolution by conducting targeted molecular analyses on a small number of 16S‐positive individuals to achieve species‐level identification within specific zoonotic genera.

Three real‐time PCRs were applied for *Francisella* species identification for all individuals detected positive for this genus using the 16S approach (*N* = 70). The ISFtu2‐qPCR allowed detecting *F. tularensis* (*i.e*., *F. tularensis* subsp. *tularensis*, subsp. *holarctica*, and *subsp. mediasiatica*), the Tul4‐qPCR enabled detecting *F. novicida* and Type B‐qPCR enabled detecting *F. tularensis* subsp. *Holarctica* [33].

A subset of *Bartonella* positive DNA extractions (*N* = 11) from four rodent species (*Clethrionomys glareolus*, *A. sylvaticus*, *C. russula*, and *G. glis*) was sequenced, to achieve a taxonomic resolution to the species level. A two‐step PCR protocol, following Galan et al. [34] was applied to amplify two genes, gltA [35] and rpoB [36]. Sequence reads were processed using the same FROGS pipeline described above.

The BioMark real‐time PCR system (Standard Biotools, San Francisco, CA, USA) was applied to 28 small mammal splenic DNA for high‐throughput microfluidic real‐time PCR for the most common bacterial tick‐borne pathogen species known to circulate or recently emerging in Europe [37, 38].

Last, we conducted additional bioinformatics analyses on the 16S rRNA metabarcoding data, focusing on the sequences from apicoplasts associated with *Apicomplexa* from the Sarcocystidae family through BLAST searches and phylogenetic tree construction. A similar species‐resolving approach was applied to the sequences of the *Mycoplasma* genus to identify those belonging to the *Mycoplasma* group (Supporting Information 1: Figure S3).

### 2.3. Statistical Analyses

We used the phyloseq package [39] for data filtering. We compiled pathogen data, keeping only presence/absence information of potentially zoonotic pathogen genera for further statistical analyses. Small mammal species with fewer than 10 individuals were excluded because of the lack of statistical power.

Statistical analyses were then conducted on the finalized dataset to assess how ecological factors shape the presence and prevalence of potentially zoonotic pathogens, which are proxies for zoonotic hazard. This later were modeled using three indices. First, the number of pathogens per individual was calculated and modeled using a Poisson distribution glmmTMB [40] to account for the high proportion of zeros in the data. Pathogen richness represents a relative measure derived from the number of taxa detected using standardized tests applied to all individuals, allowing meaningful comparisons between sites even though the screening is not exhaustive. Second, the composition of the pathogen community within individual was analyzed using the Jaccard dissimilarity matrix and a PCoA followed by a PERMANOVA test (adonis2, vegan). Only individuals carrying at least one pathogen could be considered. Last, pathogen prevalence was calculated per study site and sampling period, and analyzed using a binomial distribution with a generalized linear model (GLM) for each pathogen with a prevalence greater than 10% in the total dataset. To maximize statistical power and reduce structural zeros, only periods with confirmed pathogen presence were analyzed.

Two modeling strategies were applied to assess the influence of abiotic and biotic factors on these three indices. In the first strategy (Model 1), we included small mammal species (nine species) and sampling sites (six sites) as explanatory fixed factors. This strategy enabled to evaluate the variation in pathogen richness and prevalence at a fine spatial scale, without relying on predefined categories of species or sites. In the second strategy (Model 2), we tested specific hypotheses related to categories of species and sites with regard to adaptation to anthropization. To this end, species were grouped according to their degree of adaptation to anthropized environments (adapters, dwellers, and avoiders) and sites were categorized by their level of anthropization (protected forest, managed forest, and urban park). In both models, age class, sex, and sampling period were included as cofactors. Geographic distances between sites were not modeled explicitly because the inclusion of a site factor already accounted for spatial heterogeneity, and the limited number of sites (*n* = 6) did not provide sufficient replication to reliably estimate residual spatial autocorrelation. Nevertheless, the multivariate analysis described above (Supporting Information 1: Figure S1) enabled to confirm that the site groupings adequately represented the main spatial and ecological variation among sites.

Model selection for GLMs was performed based on AICc (MuMIn, δAICc < 2), and model fit was evaluated using DHARMa [41]. Multicollinearity between explanatory variables was assessed using the variance inflation factor (VIF > 5). No explanatory variables exceeded this threshold whatever the model considered. Differences between groups were tested using the post hoc Tukey HSD test multcomp [42] for GLMs, or the multiconstrained BiodiversityR [43] for PERMANOVA, with adjustments for multiple testing. To verify species type differences within habitats despite an unbalanced dataset, we compared species separately within the managed, protected, and urban forests subsets. When similar results were observed between protected and managed forests compared to urban ones, we grouped these two categories (protected and managed forests) to present the results more concisely and highlight differences between rural forests and urban forested parks. All scripts are available on Zenodo repository.

## 3. Results

### 3.1. Small Mammal Communities

We collected a total of 1549 small mammals, including 11 rodent species and seven Eulipotyphla species (*Soricomorpha* or *Erinaceomorpha*; Figure 1A).

The habitat type, that describes forest anthropization (Supporting Information 1: Figure S1), was the main factor influencing small mammal community composition, with PCoA axis 1 explaining 73% of the total variance. No difference was found between protected and managed forests, but composition varied significantly between urban forested parks and the two other categories of rural forests (protected and managed ones). By contrast, sampling periods primarily influenced their abundance (Figure 1B).

Small mammal species grouped significantly into urban adaptation categories (Supporting Information 1: Figure S2). Urban adapters (*Apodemus sylvaticus*, *A. flavicollis*, and *C. glareolus*) were present and dominant across all environments. Urban avoiders (*Crocidura leucodon*, *Neomys fodiens*, *Microtus agrestis*, *M. subterraneus*, and *Glis glis*) were captured only in protected and managed (rural) forests, and at relatively low abundance. In contrast, urban dwellers (*Crocidura russula*, *Microtus arvalis*, and *Erinaceus europaeus*) were trapped exclusively in urban parks.

Detailed information about these results are available in Pradel et al. [21] and in Zenodo repository.

### 3.2. Potentially Zoonotic Pathogens Detected

A wide array of pathogenic taxa has been detected, some of them being zoonotic (Table 1).

**Table 1:** Pathogens’ classification and characteristics.

Zoonotic pathogens									
Pathogen code	Kingdom	Phylum	Family	Pathogen detection and classification		Detection methods	Transmission mode	Pathogen epidemiological characteristics	
				Genus	Species			Overall prevalence	Associated disease
Borr	Bacteria	Spirochetes	Borreliaceae	*Borrelia*	*B. garinii*; *B. afzelii*; *B. miyamotoi*	16S metabarcoding; microfluidic qPCR	Vector	NA	Lyme disease
MycoZ	Bacteria	Firmicutes	Mycoplasmataceae	*Mycoplasma*	*M. penetrans*; *M. ravipulmonis*	16S metabarcoding	Direct	0.015	Respiratory infections
Fran	Bacteria	Proteobacteria	Francisellaceae	*Francisella*	*F. tularensis holarctica*	16S metabarcoding; qPCR (ISFtu2, Tul4, Type B); microfluidic qPCR	Vector^a^	0.054	Tularemia
Neoe	Bacteria	Proteobacteria	Anaplasmataceae	*Neoehrlichia*	*N. mikurensis*	16S metabarcoding; microfluidic qPCR	Vector	0.121	Tick‐borne fever
Rick	Bacteria	Proteobacteria	Rickettsiaceae	*Rickettsia*	*Rickettsia* spp.	16S metabarcoding; microfluidic qPCR	Vector	0.006	Rickettsioses
Anap	Bacteria	Proteobacteria	Anaplasmataceae	*Anaplasma*	*A. phagocytophilum*	16S metabarcoding; microfluidic qPCR	Vector	0.025	Anaplasmosis
Orie	Bacteria	Proteobacteria	Rickettsiaceae	*Orientia*	*O. tsutsugamushi*	16S metabarcoding	Vector	0.019	Scrub typhus
Lept	Bacteria	Spirochetes	Leptospiraceae	*Leptospira*	*L. kirschneri*; *L. interrogans*	qPCR (lipL32)	Direct	0.050	Leptospirosis
Lcmv	Virus	Negarnaviricota	Arenaviridae	*Mammarenavirus*	*Lcmv*‐like	Serology (IFA)	Direct	NA	Hemorrhagic fever
Hanv	Virus	Negarnaviricota	Hantaviridae	*Orthohantavirus*	*Puumala virus*; *Seoul virus*	Serology (IFA)	Direct	0.060	Hemorrhagic fever; nephropathy
Poxv	Virus	Poxviridae	Poxviridae	*Orthopoxvirus*	*Cowpox virus*‐like	Serology (IFA)	Direct	0.148	Cowpox
Bart	Bacteria	Proteobacteria	Bartonellaceae	*Bartonella*	*B. grahamii*; *B. taylori*; *B. doshiae*; *B. elizabethae*; *B*. *birtlesii;* and an unknown sp.	16S rRNA metabarcoding; 2‐step PCR (gltA, rpoB) microfluidic qPCR	Vector^a^	0.452	Bartonellosis
Chla	Bacteria	Chlamydiota	Chlamydiaceae	*Chlamydia*	*Chlamydia* spp.	16S metabarcoding	Direct	0.012	Chlamydiosis
Toxo	Protozoa	Apicomplexa	Sarcocystidae	*Toxoplasma*	*T. gondii*	16S metabarcoding	Direct	0.001	Toxoplasmosis
Eime	Protozoa	Apicomplexa	Eimeriidae	*Eimeria*	*Eimeria* spp.	16S metabarcoding	Direct	0.002	Coccidiosis
Neis	Bacteria	Proteobacteria	Neisseriaceae	*Neisseria*	*Neisseria* spp.	16S metabarcoding	Direct	0.001	Meningococci
Sarc1	Protozoa	Apicomplexa	Sarcocystidae	Unknown	Unknown	16S metabarcoding	Direct	0.106	Unknown
Sarc2	Protozoa	Apicomplexa	Sarcocystidae	Unknown	Unknown	16S metabarcoding	Direct	0.025	Unknown

*Note*: It presents the various pathogens, including bacteria, viruses, and protozoa, that have been detected in this study. Taxonomic details are provided (kingdom, phylum, family, genus, and species). The mode of transmission is specified as direct or vector‐borne, with direct transmission encompassing physical contact or environmental exposure. The overall prevalence or seroprevalence presented in this study was calculated as a proportion of positive individuals, with values ranging from 0 to 1. The principal and potential known diseases associated with each pathogen are indicated.

^a^Indicates that other modes of transmission are known for these potentially vector‐borne pathogens.

Serological assays revealed antibodies against four virus families. IFAT detected antibodies against *Orthohantavirus*, and molecular sequencing confirmed the presence of PUUV in bank voles from Jura region [9] and Seoul virus in brown rats from urban parks [44]. Low seroprevalence was noted for PUUV antibodies in house mice (1.17%) and DOBV in Apodemus species (<0.20%) and *C. russula* (1.04%). We attempted to detect hantavirus in the lungs of these seropositive individuals, but were unsuccessful, supporting the conclusion of incidental exposure in these mice and insectivores [45]. A high seroprevalence was observed for *Orthopoxvirus* (13.19%) across all sites and species, except for *Crocidura* sp. and *Glis glis. Mammarenavirus* seroprevalence was low (1.14%). However, no testing was conducted on samples collected in autumn 2021 samples.

qPCR analyses identified pathogenic *Leptospira* bacteria in all small mammal hosts, with the exception of *Glis glis* and *Crocidura leucodon*. The overall prevalence was 0.05 (Table 1). consistent with their broad host‐spectrum reported in the literature. Genotyping in a subset of individuals previously revealed a high diversity of *Leptospira* species in urban parks [29]. In the rural forests studied here, *L. kirschneri Grippotyphosa* and *L. interrogans Australis* were detected in *C. glareolus* and *A. flavicollis*. These species and serogroups were also detected in the urban parks.

The 16S rRNA metabarcoding approach was applied to 1284 individuals. It enabled to detect nine bacteria genera and four protists (Apicomplexa) that are potentially pathogenic for humans and animals (Table 1). Some taxa are obligate vector‐borne: *Neoehrlichia*, *Borrelia*, *Rickettsia*, *Anaplasma*, and *Orientia*, while others are or may be spread through direct (*Mycoplasma*, *Bartonella*, and *Francisella*), potentially sexual (*Chlamydia*) or oral (Sarcocystidae, *Eimeria*, and *Toxoplasma*) transmission routes.

qPCR assays confirmed the presence of *Francisella tularensis holarctica* in all 70 individuals that tested positive using the metabarcoding approach. This bacterium is the causative agent of tularemia, a high‐risk disease in Europe. The sequencing of the rpoB and gltA genes enabled to identify *Bartonella grahamii* (zoonotic), *B. taylorii*, *B. doshiae*, *B. gliris*, *B. birtlesii*, and an unknown *Bartonella* species.

The high‐throughput real‐time PCR approach also enabled to identify *Neoehrlichia mikurensis*, *Anaplasma phagocytophilum*, *Borrelia garinii*, *B. afzelii*, and *B. miyamotoi*.

Phylogenetic analyses using the 16SV4 rRNA sequences identified zoonotic variants of *Mycoplasma*, including *M. penetrans* and *M. ravipulmonis* and four taxa groups from the Sarcocystidae family, with some of the OTUs clustering with *Eimeria* sp. and *Toxoplasma gondii* (Supporting Information 1: Figure S3).

### 3.3. Drivers of Zoonotic Pathogen Presence and Transmission Dynamics

#### 3.3.1. Richness of Zoonotic Pathogens per Individual

Pathogen richness varied significantly across study sites, periods, and small mammal species (Model 1: zero‐inflated Poisson, Wald test: sites: *χ* ^2^ = 44.79, *p* = 1.6 × 10^−8^; periods: *χ* ^2^ = 33.53, *p* = 9.3 × 10^−7^; species: *χ* ^2^ = 159.60, *p* < 2.2 × 10^−16^; AIC = 2968; Supporting Information 1: Figure S4). Considering habitat and species categories (Model 2: AIC = 3178), we found a significant influence of forest anthropization (zero‐inflated Poisson, Wald test: *χ* ^2^ = 7.25, *p* = 2.7 × 10^−3^) and small mammal urban adaptation (zero‐inflated Poisson, Wald test: *χ* ^2^ = 55.70, *p* = 8.0 × 10^−3^). These two models are therefore complementary: Model 1 provides a detailed quantification of individual‐level variation, whereas Model 2 highlights broader ecological trends across habitats and species types.

Considering habitat types, pathogen richness per individual was lower in urban parks than in managed forests (post hoc Tukey HSD, *β* = −0.17, *z* = −2.6, *p* = 0.02; Figure 2A), but contrasted patterns were detected for the protected forests FRFGRI and FRFGLA (Supporting Information 2: Table S1 and Supporting Information 1: Figure S4A). This pattern was still significant when considering adapter species only, that is, species that can settle in all forested habitats whatever their level of anthropization. Indeed, for adapter species, pathogen richness was lower in urban forests compared to managed forests (zero‐inflated Poisson, urban parks—managed forests, *β* = −0.17, *z* = −2.60, *p* = 0.03; Figure 2C), but not significantly different from protected forests (zero‐inflated Poisson, managed—protected forests, *β* = 0.03, *z* = 0.40, *p* = 0.92; urban parks—protected forests, *β* = −0.14, *z* = −1.68, *p* = 0.21).

Figure 2Predicted pathogen richness from glmmTMB models (A) across habitat types (protected forests in light green, managed forests in yellow, and urban parks in orange), (B) by species urban adaptation categories (avoiders in yellow, adapters in dark green, and dwellers in red), and (C) across habitat types for adapter species only. Each point represents an individual. Lowercase letters indicate statistically similar or different groups based on post hoc comparisons. Groups sharing the same letter are not significantly different from each other.

**Figure:**
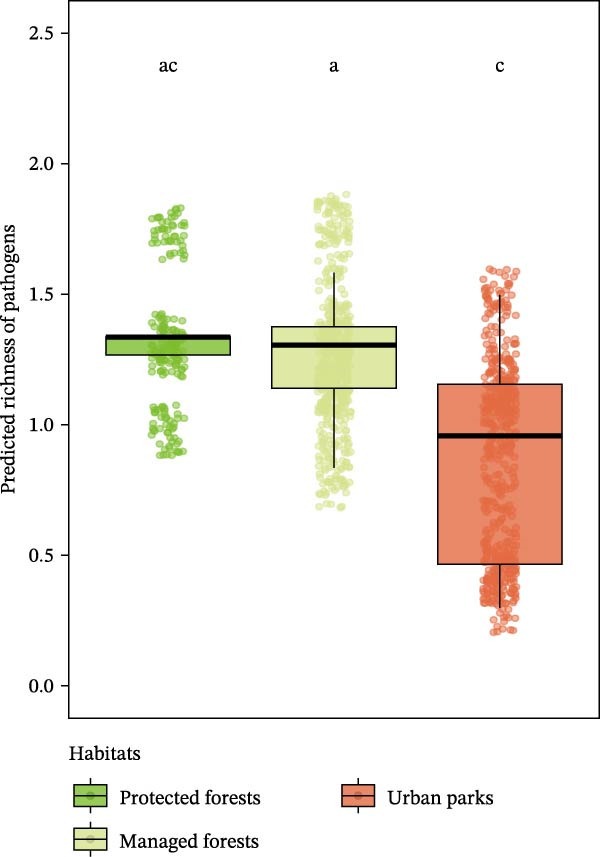
(A)

**Figure:**
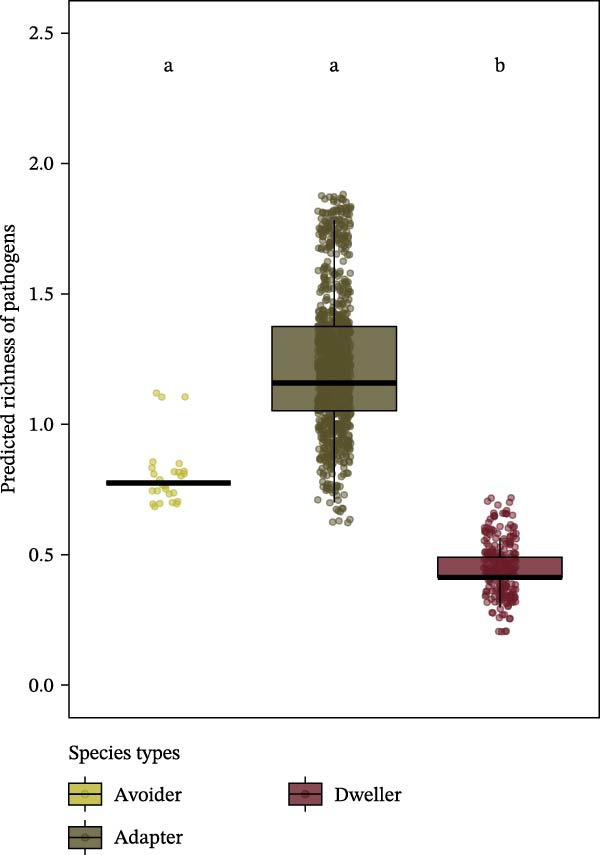
(B)

**Figure:**
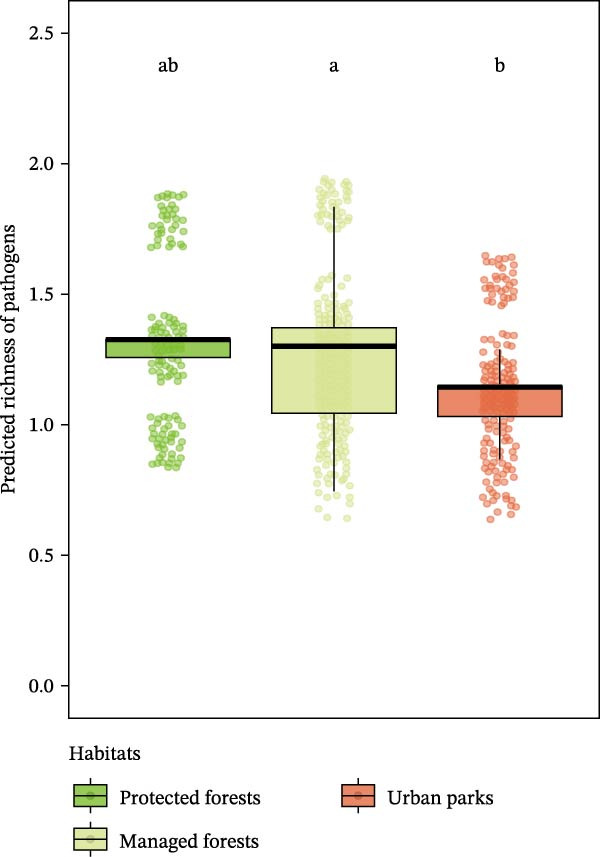
(C)

Considering species urban adaptation categories, urban dweller species exhibited significantly fewer pathogen richness than urban adapter or avoider species (post hoc Tukey HSD: adapter—dweller, *β* = −0.86, *z* = −7.48, *p*_adj_ < 10^−3^; dweller—avoider, *β* = −0.77, *z* = −2.98, *p*_adj_ = 7 × 10^−3^; Figure 2B and Supporting Information 2: Table S2). Adapter species harbored about two to three times more zoonotic pathogens than other species categories (Figure 2B). These patterns held even when species co‐occurred in the same habitat types (Supporting Information 2: Table S2, Supporting Information 1: Figure S5), although this result was significant in urban parks only (zero‐inflated Poisson, dweller (Ref: adapter): *β* = −0.85, *z* = −7.28, *p* < 10^−3^).

Sampling period significantly influenced pathogen richness per individual, with a significant increase detected from spring 2020 to autumn 2021, and a significant decline in spring 2022 (post hoc tests are detailed in Supporting Information 2: Table S1; Supporting Information 1: Figure S4B).

Among host‐related factors, age class significantly influenced pathogen richness, with adults harboring approximately 37% more zoonotic taxa than juveniles (zero‐inflated Poisson, *β* = 0.32, *z* = 5.16, *p* < 10^−3^).

#### 3.3.2. Composition of the Pathogen Community Within Individuals

PERMANOVA showed that the pathogen community composition within individuals varied significantly according to sampling periods (*R^2^* = 0.04, *p* = 10^−3^), study sites (*R^2^* = 0.07, *p* = 10^−3^), host species (*R^2^* = 0.17, *p* = 10^−3^), and age class (*R^2^* <0.4%, *p* = 2 × 10^−3^). Despite their significance, no single factor structured individual groups according to their pathogen composition, reflecting strong inter‐individual heterogeneity shaped by host‐ and context‐specific pathogen profiles (Supporting Information 1: Figure S6 and Supporting Information 2: Table S3).

**Figure 3:**
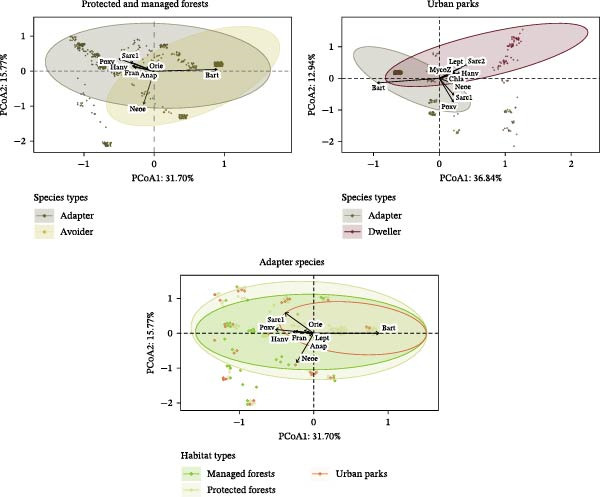
Pathogen composition shown by PCoA based on Jaccard matrix (presence/absence), with points representing individuals (some may overlap). Ellipses (90% threshold) represent species urban adaptation categories in each habitat type (protected and managed forests combined due to similar composition) or habitat types. Environmental vector fitting analysis was performed using envfit. Pathogens that were significantly associated with community composition (*p*_adj < 2 × 10^−3^) are indicated by arrows, showing direction and strength of the signal. Pathogens codes: MycoZ = *Mycoplasma zoonotic*, Fran = *Francisella*, Neoe = *Neoehrlichia*, Rick = *Rickettsia*, Anap = *Anaplasma*, Orie = *Orientia*, Lept = *Leptospira*, Hanv = *Orthohantavirus*, Poxv = Orthopoxvirus, Bart = *Bartonella*, Chla = *Chlamydia*, Sarc1 = Sarcocystidae1, Sarc2 = Sarcocystidae2.

Clearer patterns emerged when individuals were grouped based on anthropization‐related factors (Supporting Information 1: Figure S7A). Small mammals from managed and protected forests exhibited similar, though weakly differentiated, pathogen communities (SumOfSqs = 3.62, *p* = 1.69, *p* = 10^−3^), whereas individuals from urban parks showed markedly distinct profiles compared to both forest types (urban‐managed: SumOfSqs = 6.45, *p* = 2.74, *p* = 10^−3^; urban‐protected: SumOfSqs = 4.97, *p* = 2.62, *p* = 0.001), as illustrated by the clear separation of habitat types in the PCoA ordination (Supporting Information 1: Figure S7A). A similar result was observed when analyzing the composition of pathogens community for adapter small mammals only (*R* ^2^ = 0.04, *p* = 0.001; Figure 3 and Supporting Information 2: Table S4), with individuals from urban parks displaying markedly distinct profiles compared to both forest types. In brief, *Bartonella* was more prevalent in urban parks compared to rural forests, whereas the opposite pattern was detected for *Orthopoxvirus* (Supporting Information 2: Table S5).

Pathogen communities differed between host urban adaptation categories (PERMANOVA, *R^2^* = 0.03, *p* = 10^−3^), with the strongest divergence observed between urban adapter and urban dweller species (Supporting Information 1: Figure S7B and Supporting Information 2: Table S4), even within the same habitat (urban parks: adapter–dweller: *R^2^* = 0.12, *p* = 10^−3^; rural forests [managed and protected] adapter–avoider: *R^2^* = 0.005, *p* = 0.01; Figure 3).

Because of the absence of significant patterns between protected and managed forests, and to assess differences in species composition within habitats within an unbalanced dataset, we conducted separate analyses for rural (managed and protected) and urban forest datasets. PCoA biplots highlighted several key pathogens contributing to these variations in pathogen community composition (Figure 3). More specifically, in rural forests, adapter species were associated with higher contributions of *Orthopoxvirus*, *Orthohantavirus*, Sarcocystidae group 1, and *Francisella* sp., and lower contributions of *Orientia* sp. and *Anaplasma* sp., compared to avoiders. Their pathogen communities were strongly shaped by *Bartonella* sp., which exerted a dominant structuring influence. In urban parks, dwellers differed from adapter species by higher occurrences of Sarcocystidae group 2, *Orthohantavirus*, *Leptospira* sp., and zoonotic *Mycoplasma* sp. Adapter species exhibited higher occurrences of *Bartonella* sp.

#### 3.3.3. Prevalence of Zoonotic Pathogens

The prevalence of zoonotic pathogens detected in this study varied markedly in space, time and between small mammal species (Figure 4A,B; see details in Zenodo repository). Further analyses focused on *Bartonella* sp., *Orthopoxvirus*, *Neoehrlichia* sp. and Sarcocystidae group 1, *Francisella turensis*, *Leptospirosa* sp. and *Orthohantavirus*, all of which showed seroprevalence exceeding 10% (Table 1).

Figure 4Heatmap of (sero) prevalence of pathogens: (A) by sampling sites ordered according to the level of anthropization (protected forests: light green; managed forests: yellow; urban parks: orange) and (B) by small mammal species, grouped by urban adaptation categories (adapters: green‐gray; avoiders: light green; urban dwellers: red). Color gradients from light blue to dark purple indicate seroprevalence values. Vertical bar charts on the left show the number of zoonotic pathogens detected per site (A) and per host species (B). Inverted horizontal bar charts below indicate the number of sites (A) or host species (B) associated with each pathogen. Pathogens are ordered by prevalence level, respectively, per site and host species. Dark gray bars represent obligate vector‐borne transmission, light gray bars indicate other potential modes of transmission (via direct, sexual contact, oral routes and/or the environment). Species code: *Asyl* = *Apodemus sylvaticus*, *Afla* = *Apodemus flavicollis*, *Cgla* = *Clethrionomys* (*syn. Myodes*) *glareolus*, *Crus* = *Crocidura russula*, *Cleu* = *Crocidura leucodon*, *Mmus* = *Mus musculus*, *Rnor* = *R norvegicus*, *Ggli* = *Glis glis*, *Marv* = *Microtus arvalis*. Site codes: FRPLTO = Parc de la Tête d’Or, Rhône region; FRPDLL = Lacroix Laval, Rhône; FRFCOR = Cormaranche, Ain; FRFMIG = Mignovillard, Jura; FRFGRI = Griffe du Diable, Arvière Ain; FRFGLA = La Glacière, Jura.

**Figure:**
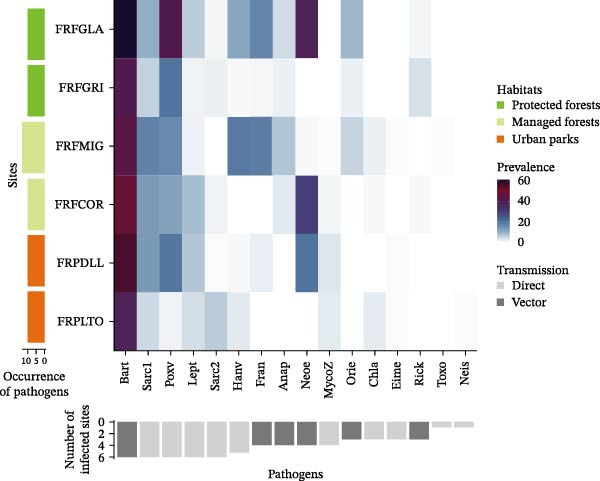
(A)

**Figure:**
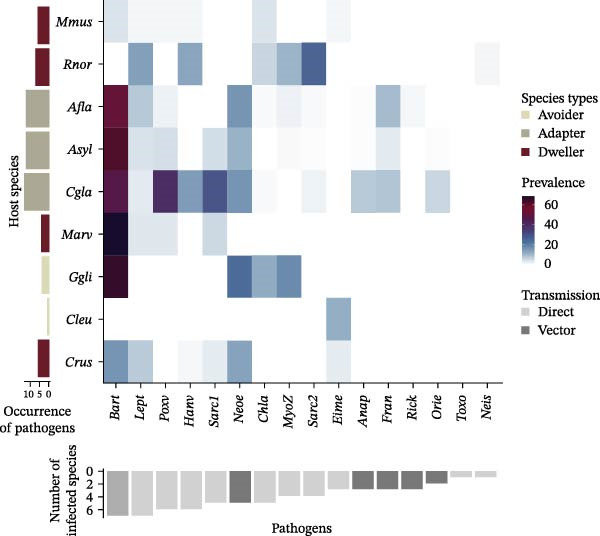
(B)

*Bartonella* sp. infection, which involved a broad host range, varied with host species (ANOVA, Model 1, *β* = 268.66, *p* = 2.2 × 10^−16^). Five species were predominantly infected (*Glis glis*, *Microtus arvalis*, *Apodemus sylvaticus*, *A. flavicollis*, and *C. glareolus*; prevalence >40%; Figure 4B), whereas *M. musculus* and *R. norvegicus* had very low prevalence (<5%; post hoc, Supporting Information 2: Table S5). On average; dwellers showed a threefold lower prevalence than adapter and avoider species (Model 2, post hoc: *β* = –2.99, *p* < 10^−4^; *β* = –3.24, *p* < 10^−4^, respectively). Prevalence also varied among sites (*β* = 46.95, *p* = 5.8x10^−9^) and sampling periods (*β* = 42.43, *p* = 1.4 × 10^−8^), being higher in urban habitats than in managed forests (*β* = 0.98, *p* < 10^−3^) or protected forests (*β* = 0.66, *p* = 2.7 × 10^−3^; Figure 4A). Temporal peaks occurred in spring 2020 and autumn 2021 (Supporting Information 1: Figure S8 and Supporting Information 2: Table S5).

Antibodies against OPXV also showed strong spatial, temporal and species‐related variation (ANOVA, Model 1: sites, *χ* ^2^ = 11.70, *p* = 0.04; species, *χ* ^2^ = 249.05, *p* = 2.2 × 10^−16^; periods, *χ* ^2^ = 82.839, *p* = 2.2 × 10^−16^). All small mammal species except *Crocidura* sp. and *G. glis* were seropositive. Urban species categories and habitat types significantly explained seroprevalence (ANOVA, Model 2: species urban categories: *χ* ^2^ = 37.46, *p* = 7.36 × 10^−9^; habitats types: *χ* ^2^ = 13.33, *p* = 10^−3^). Dwellers showed almost threefold lower seroprevalence than urban adapters (post hoc test, est. = −2.86, *p* = 2 × 10^−4^). Protected forests had higher prevalence than other habitats (protected—managed forests: est. = 0.56, *p* = 2.5 × 10^−3^; urban parks—protected forests: est. = −0.88, *p* = 10^−3^). A peak in seroprevalence occurred in autumn 2021 (Supporting Information 1: Figure S8 and Supporting Information 2: Table S5).

The prevalence of Candidatus *Neoehrlichia* varied significantly between sites (*χ* ^2^ = 257.84, *p* = 2.2 × 10^−16^), with higher infection rates in the rural forests FRFGLA and FRFCOR than in the urban park FRPDLL (Figure 4A and Supporting Information 2: Table S5). Habitat type had no significant effect (*χ* ^2^ = 5.51, *p* = 0.06). Species categories, however, explained a significant part of the variation (Model 2: *χ* ^2^ = 13.1, *p* = 10^−3^). Dwellers had almost twice lower prevalence than urban avoider and adapter species (post hoc test, dweller—adapter: *β* = −1.52, *p* = 6 × 10^−3^; dweller—avoider: *β* = −1.84, *p* = 0.04). Temporal variation was also significant (*χ* ^2^ = 30.42, *p* = 4.03 × 10^−6^), with lower prevalence in autumn 2021 (Supporting Information 1: Figure S8 and Supporting Information 2: Table S5).

Finally, the prevalence of Sarcocystidae 1 varied among species and across space and time (ANOVA: sites, *χ* ^2^ = 17.56, *p* = 4 × 10^−3^; species, *χ* ^2^ = 204.25, *p* = 2.2 × 10^−16^; periods, *χ* ^2^ = 32.06, *p* = 1.8 × 10^−6^). *Clethrionomys glareolus* showed markedly higher infection levels than other species (Figure 4B and Supporting Information 2: Table S5). No clear pattern emerged with respect to habitat type or species categories.

## 4. Discussion

### 4.1. A Wide Diversity of Zoonotic Hazard

Zoonoses associated with small mammals are a global concern, particularly in tropical regions and urban environments where high abundance or diversity of reservoir species, intensified anthropogenic pressures, and ecological or socioeconomic conditions favor pathogen transmission to humans [2, 3, 46]. Our analysis highlights a substantial and previously underestimated circulation of rodent‐borne pathogens in Eastern France, affecting a diversity of habitats regardless of their level of anthropization.

By combining multiple pathogen detection approaches, we confirmed the presence of known zoonotic agents—such as PUUV, *Leptospira* sp., *Borrelia* sp., and *Francisella tularensis* in Eastern France, all of which being already known from wildlife monitoring [9, 29, 33, 47] and human clinical cases (see reports from respective CNR). A more detailed investigation of *Leptospira* in these rural forests revealed the circulation of L. *kirschneri* Grippotyphosa and *L. interrogans* Australis. These findings provide essential insights for the development of targeted vaccination strategies for protecting professionals with high occupational exposure, such as forest workers, particularly since the currently available vaccine only covers *L. icterohaemorrhagiae*. They underscore the need for a surveillance‐to‐action approach, enabling the identification of locally circulating strains and informing the development of targeted vaccines or the implementation of appropriate sanitary and management measures to effectively reduce exposure risk.

Our study also reinforces existing knowledge on the circulation of pathogens that had previously been documented exclusively through wildlife surveillance, including Seoul virus in Lyon [44], *Orthopoxvirus* or *Mammarenavirus* in the Jura region [48].

Importantly, our findings highlight an overlooked threat with regard to the high diversity of vector borne‐bacteria detected in ticks (*Neoehrlichia mikurensis*, *Rickettsia* sp., and *Anaplasma phagocytophilum*) and mites (*Orientia tsutsugamushi*). This diversity of vector‐borne bacteria underscores the need to expand human diagnostic frameworks beyond *Borrelia* sp. alone, allowing for more accurate recognition of the full spectrum of tick‐borne infections that may underlie the wide range of symptoms currently attributed to Lyme disease [49]. Historically and more recently, other vectors, such as fleas, have been responsible for transmitting major pathogens (e.g., *Yersinia pestis*, *Bartonella* sp., and *Rickettsia* sp [50]). These observations underscore the importance of integrated, multihost and multipathogen surveillance, using broad, unbiased detection methods such as metabarcoding [26], to accurately assess and mitigate zoonotic hazards, while recognizing that qPCR approaches remain more sensitive for single‐target detection and low‐abundance organisms.

While comprehensive, our investigation likely remains incomplete. Important taxonomic groups such as protozoa (e.g., *Giardia* sp. and *Babesia* sp.), helminths (e.g., *Echinococcus multilocularis*), and viruses (e.g., flaviviruses and coronaviruses) were not targeted and may represent additional, unrecognized sources of zoonotic hazard. Furthermore, some of the potentially zoonotic taxa we detected, such as *Streptobacillus* sp., *Rickettsia* sp., and Sarcocystidae, warrant further investigation to determine whether they include species of direct relevance to human health.

### 4.2. What Shapes Zoonotic Hazard?

Our study reveals that small mammal species is a primary factor shaping the diversity and prevalence of zoonotic pathogen communities across forest types representing different levels of anthropization. This finding corroborates previous studies showing that small mammal species exhibit varying levels of reservoir competence [11]. It also emphasizes the significance of host–pathogen specificity, which may be driven by phylogenetic relationships, but also by host traits that influence susceptibility, pathogen exposure, and transmission rates. Among them, immunity, behavior, or ecological niche have been widely studied [14, 51–53].

Here, we found that urban adapter species, which are generalist species capable of inhabiting a wide range of environments including anthropogenic habitats, harbor zoonotic pathogen communities that exhibit higher richness compared to those of habitat specialists (either urban dwellers or avoiders). Notably, these differences persist even within the same habitat type. This finding aligns with recent meta‐analyses suggesting that generalist species tend to allocate more resources in rapid growth and reproduction over immune investment, especially in variable or unpredictable environments, potentially resulting in higher reservoir competence for pathogens [11, 14, 54, 55]. However, these results have to be considered cautiously as avoider species were represented by a limited number of individuals, due to their low abundance and/or avoidance behavior, while dweller species were difficult to capture and were successfully trapped at a single site only (Lyon urban park). This restricted sampling may introduce bias and limit the representativeness of urban dweller’s zoonotic pathogen communities.

Our analysis also underscores that habitat type, here categorized in terms of anthropogenic disturbance, also strongly influences zoonotic pathogen communities. This was mainly observed when comparing urban and rural forests, as managed and protected forests exhibited similar patterns of richness and composition of pathogen community. This pattern was also detected when focusing on urban adapter species only, emphasizing the role of habitat filtering and ecological constraints on pathogen presence and transmission. We highlighted cascading effects of forest anthropization on the composition, diversity and interactions of small mammals and zoonotic pathogen communities. These shifts operate through multiple ecological mechanisms, including altered ecological niches, and changes in abiotic conditions and interspecific interactions [52, 56].

Although several studies have revealed higher small mammal specific richness in undisturbed habitats compared to anthropogenic ones, due to the exclusion of more specialized, disturbance sensitive species [57], our results rather underscore differences in the composition of these hosts communities. Avoider species were detected in rural forests, both protected and managed ones; dweller species were detected in urban parks, and urban adapter species were trapped in all types of habitats. These findings align with growing evidence that urban parks may host diverse communities dominated by generalist and synanthropic species, characterized by high ecological plasticity, rapid reproduction rates and behavioral adaptability [58–60]. We also showed that urban adapter species thrive in urban parks (e.g., *A. sylvaticus* and *C. russula*), reaching high densities due to predator release and abundant anthropogenic resources [56, 61–63].

These shifts in host community structure, coupled with stressful urban conditions (chemical pollutants and excess artificial light) known to impair immune function and increase host susceptibility, are expected to elevate parasite richness, including zoonotic ones, in urban environments.

Contrary to these expectations, our findings indicate that zoonotic pathogen richness was lower in urban parks compared to rural forests, and not all pathogens exhibited higher prevalence in urban parks. Broader sampling beyond parks might yield different results, but several factors could explain this pattern. First, urban‐adapted mammal species are not necessarily linked to more zoonotic parasites (see impact of publication bias [16]), emphasizing the need for more field‐based data. Second, the constant availability of anthropogenic food in urban parks may decrease foraging and exposure to environmentally transmitted parasites [64]. Third, urban‐induced changes in microclimate and vegetation, such as reduced canopy cover and increased forest fragmentation, may limit the diversity or abundance of vectors and intermediate hosts [65]. A recent meta‐analysis supports this, showing that parasites with complex life cycles were less prevalent in urban dwelling primate and carnivores compared to less anthropized habitats, in line with the urban refuge hypothesis [60]. Similarly, our study showed a decline of vector‐borne zoonotic pathogens such as *Rickettsia* sp., *Anaplasma* sp., and *Orientia* sp. in urban parks, though *Neoehrlichia mikurensis* was an exception, emphasizing the complex and multifaceted impacts of forest anthropization on zoonotic hazards.

Our results also suggest that specific ecological interactions between hosts and pathogens may play a key role in determining how anthropization influences zoonotic hazard, leading to idiosyncratic patterns. For example, *Bartonella* sp. were highly prevalent across host species and sites, with higher prevalence in urban adapter species, and in urban parks. This pattern may reflect environment‐driven changes in host behavior, exposure or susceptibility, though this trend was not observed for other pathogens, suggesting that *Bartonella*’s ecology plays an important role. Factors like *Bartonella* species diversity, coinfections [66], or environment‐dependent transmission [67] may be involved. These findings highlight the complexity of host–pathogen dynamics in anthropized landscapes and the need to consider pathogen‐specific traits in zoonotic hazard assessments.

Differences between protected and managed rural forests appeared more subtle than expected, potentially due to confounding ecological and climatic characteristics. Shared abiotic features could explain the similarity of host communities’ composition. Both forest types were dominated by urban adapters, with low abundance of avoiders. Moreover, site‐specific characteristics may blur distinctions: for example, ’La Glacière’ reserve is small and bordered by managed forests, likely experiencing similar pressures than the close managed forests of Mignovillard. Still, some pathogens showed varying prevalence. *Chlamydia* sp., zoonotic *Mycoplasma* sp., and PUUV tended to be less prevalent in protected forests [9], while *Orthopoxvirus* seroprevalence was higher—possibly due to greater biodiversity or older host populations increasing exposure [68].

More broadly, the spatial arrangement of our sampling sites reflected ecological constraints. Urban parks are necessarily located within cities, which are highly fragmented and largely devoid of extensive forested areas. As a result, the urban sites sampled were close to one another but geographically distant from the rural forests. In contrast, the protected and managed rural sites could be paired because of their relative proximity and similar ecological conditions. In the future, expanding the study to include additional urban parks representing a broader range of ecological contexts will help assess the generality of our conclusions.

Last, fine‐scale spatial and temporal variations in zoonotic pathogen prevalence were observed, with particular taxa being detected only once during this multi‐annual survey (e.g., *Francisella tularensis*). Local abiotic conditions, mast seeding, may boost small mammal abundance and in turn, the transmission rate of certain pathogens (e.g., for *Orthohantavirus* [69, 70]). These findings underscore the need for longitudinal and spatially resolved studies to capture transient yet ecologically meaningful shifts in zoonotic hazard, particularly in the context of ongoing environmental changes.

In this study, pathogens were analyzed at the genus level, to provide a community‐wide perspective on zoonotic hazard patterns. However, differences among pathogens, such as transmission routes, host specificity, or ecological requirements, may shape their responses to anthropization. Addressing these aspects in future studies, using larger and more diverse datasets, will be essential to refine our understanding of pathogen–host–environment relationships.

### 4.3. Consequences for Zoonotic Hazard Mitigation

The observed spatial and temporal heterogeneity in zoonotic pathogen richness and prevalence across rural forests and urban parks calls for tailored surveillance strategies and context‐specific public health responses.

Several zoonotic pathogens, such as *Leptospira* sp., *Orthohantavirus* (SEOV), and *Bartonella* sp., were found at high prevalence in urban wildlife, particularly in adapter species such as *Apodemus sylvaticus* and *Rattus norvegicus*. These species thrive in close proximity to humans, providing opportunities for exposure and potential spillover risk despite lower overall pathogen richness in urban parks compared to rural forests. Urban parks, where high human densities and activity intersects with abundant competent reservoir hosts, therefore represent critical zoonotic interfaces [71]. With expanding urban greening initiatives, proactive measures including surveillance and targeted prevention strategies will become increasingly important [72].

Surveillance should focus on sentinel species, particularly synanthropic adapters, that serve as main competent hosts for key zoonotic pathogens [59], and incorporate integrated rodent management strategies. These include improved waste management and physical buffers separating human recreational areas from rodent habitats. Interventions should be concentrated in areas where exposure or contacts between human or captive animal and infected rodents are likely and frequent. Altogether, these proposals should provide local environmental and Public health stakeholders with science‐based tools for implementing evidence‐driven interventions [73].

In rural forests, a considerable diversity of rodent‐borne zoonotic pathogens, in particular vector‐borne ones, was detected. Even though human and domestic animal exposure may be lower, the need for continuous surveillance, public education and forest management are needed to reduce human–wildlife contact and support ecosystem health. Monitoring should capture seasonal and annual fluctuations in host populations and pathogen circulation, forming an early warning system for emerging threats. Effective surveillance must be large‐scale to assess the diversity of host and vector species. This should include noninvasive wildlife survey (e.g., camera traps and environmental DNA), environmental data (e.g., temperature and humidity), and pathogen screening.

## 5. Conclusion

This study reveals a high diversity of pathogens circulating within small mammal communities across both urban and rural forests in Eastern France. Zoonotic hazard emerges from complex interactions between host species traits and the degree of habitat anthropization. These results highlight the need for developing habitat‐specific research and surveillance programs that consider seasonal fluctuations, host ecology, and environmental changes. Consequently, fostering strong collaborations between researchers, environment managers, clinicians, and public health authorities will be critical to anticipate, detect, and reduce zoonotic hazard and to minimize human exposure in increasingly human‐altered landscapes.

## Funding

This research was funded through the 2018−2019 BiodivERsA joint call for research proposals, under the BiodivERsA3 ERA‐Net COFUND programme, and with the funding organization ANR (France).

## Ethics Statement

The CBGP laboratory has approval (F‐34‐169‐001) from the Departmental Direction of Population Protection (DDPP, Hérault, France) for the sampling of small mammals and the storage and use of their tissues. All procedures related to small mammals captured in this study complied with the ethical standards of the relevant national and European regulations on the protection of animals used for scientific purposes (Directive 2010/63/EC revising Directive 86/609/EEC, adopted on 22 September 2010). All procedures have undergone validation by the regional ethics committee “Comite d’Ethique pour l’Expérimentation Animale Languedoc Roussillon n˚36” in 2020 (Ref: 2020‐02‐v2).

## Conflicts of Interest

The authors declare no conflicts of interest.

## Supporting Information

Additional supporting information can be found online in the Supporting Information section.

## Supporting information


**Supporting Information 1** Figure S1: Principal component analysis (PCA) of study sites based on their biogeoclimatic (A) and anthropogenic (B) characteristics. Figure S2: Principal coordinates analysis (PCoA) based on the distribution of small mammal species across study sites. Figure S3: Phylogenetic trees of Sarcocystidae and *Mycoplasma* based on 16S V4 sequences. Figure S4: Boxplots of predicted individual pathogen richness from zero‐inflated Poisson GLMs, according to: (A) sites, ordered according to the level of anthropization; (B) periods; (C) small mammal species. Figure S5: Boxplots of predicted individual pathogen richness from zero‐inflated Poisson GLMs, according to the ecological types of small mammals and the level of anthropization. Figure S6: Pathogen community composition (presence of at least one pathogen among the 16 tested) based on Jaccard dissimilarities visualized by PCoA, colored according to site, sampling period, and host species. Figure S7: Pathogen community composition (presence of at least one of the 16 pathogens) based on Jaccard dissimilarities. Figure S8: Heatmap of pathogen (sero)prevalence across sampling periods. Sampling periods are ordered chronologically.

**Supporting Information 2** Table S1: Richness of pathogen taxa per individual according to species and sites (Model 1). Table S2: Richness of pathogen taxa per individual according to species categories and habitat types (Model 2). Table S3: PERMANOVA showing differences in community composition (Jaccard distance) according to sites and species (Model 1). Table S4: PERMANOVA showing differences in community composition (Jaccard distance) according to habitat types and species categories (Model 2). Table S5: GLM analyses of pathogen prevalence according to sites and species (Model 1) and habitat types and species categories (Model 2).

## Data Availability

16S data from splenic DNA are available at https://zenodo.org/records/12518286. Pathogen characterization data and scripts are available at https://10.5281/zenodo.15671364.
